# Quantifying the Colonization of Environmental Microbes in the Fish Gut: A Case Study of Wild Fish Populations in the Yangtze River

**DOI:** 10.3389/fmicb.2021.828409

**Published:** 2022-02-17

**Authors:** Haile Yang, Jinming Wu, Hao Du, Hui Zhang, Junyi Li, Qiwei Wei

**Affiliations:** Key Laboratory of Freshwater Biodiversity Conservation, Ministry of Agriculture and Rural Affairs of China, Yangtze River Fisheries Research Institute, Chinese Academy of Fishery Sciences, Wuhan, China

**Keywords:** gut microbiota, wild fish population, natural water, Yangtze River, environmental microbiota

## Abstract

In aquatic animals, gut microbial communities shift with host development and living environments. Understanding the mechanism by which the environment impacts the gut microbial communities of aquatic animals is crucial for assessing and managing aquatic ecosystem health. Here, we proposed a simplified framework for the colonization and dynamics of gut microbial communities. Then, to quantify the colonization of environmental microbes in the wild fish gut, the current study used 16S rRNA gene amplicon sequencing to obtain the structure of the water environmental microbial community and the gut microbial community in 10 wild fish populations (*Leiocassis crassilabris*, *Leiocassis longirostris*, *Pelteobagrus vachelli*, *Silurus asotus*, *Siniperca chuatsi*, *Coilia brachygnathus*, *Aristichthys nobilis*, *Hypophthalmichthys molitrix*, *Coreius heterodon*, and *Xenocypris argentea*) from the Wuhan section of the Yangtze River, and the relationship of these microbial communities was analyzed. The results identified that in most individuals, approximately 80% of gut microbes [at the operational taxonomic unit (OTU) level] were shared with the water environmental microbial community (except for individuals of *Siniperca chuatsi* and *Coilia brachygnathus*, approximately 74%). In approximately 80% of individuals, more than 95% of microbial species (OTUs) in the gut were transient. For fish species, more than 99% of microbial species (OTUs) that were introduced into the gut were transient. Nearly 79% of OTUs and 89% of species of water environmental microbes could be introduced into the fish gut. Driven by the introduction of transient microbes, fishes with similar feeding habits had similar gut microbial communities. The results indicated that for adult wild fishes, most gut microbiota were transient from the environmental microbiota that were related to fish feeding habits. We therefore encourage future research to focus on environmental microbiota monitoring and management to promote the better conservation of aquatic animals. It was important to note that, because of various influence factors, interspecific differences and individual variations on gut microbial community characteristics, the quantification of gut microbes in the current work was approximate rather than accurate. We hope that more comparable research could be conducted to outline the quantitative characteristics of the relationship between gut microbial community and aquatic environment microbial community as soon as possible.

## Introduction

Gut microbial communities of fish shift with host development and living environments (Yan et al., 2016; Lokesh et al., 2018; Derome and Filteau, 2020). Water is a living matrix in which many microbes reside, and it serves as a source for the fish gut microbiome (Sun et al., 2019; Sehnal et al., 2021). Initially, fish embryos develop in a relatively constant bacteria-free environment (i.e., within the egg), although after spawning, some environmental microbes quickly colonize the egg surface from the surrounding water (Butt and Volkoff, 2019; Legrand et al., 2020). After hatching, environmental microbes colonize the gut of larvae through the ingestion of water (Giatsis et al., 2015; Abdul Razak et al., 2019). After the first feeding, new microbial communities are introduced into the gut with the diet, leading to an increase in microbial diversity (Romero and Navarrete, 2006; Giatsis et al., 2015). The gut microbiota further shifts with host development and with changes in diet (Li et al., 2017; Wilkes Walburn et al., 2019). At an early stage, the gut microbiota is influenced mainly by the introduction of environmental microbes with water and diet; however, as the immune system and nutrition metabolism develop, gut microbes are selected and enriched gradually (Yan et al., 2016; Li et al., 2017; Lokesh et al., 2018; Zhang et al., 2018; Wilkes Walburn et al., 2019; Yang et al., 2020). Thus, gut microbial community succession can be clearly separated according to host developmental stages from larvae to adults (Lokesh et al., 2018; Xiao et al., 2021). In adult fish, the gut microbiota can be divided into two groups: resident and transient microbial communities (Ringø et al., 2016; Legrand et al., 2020). Resident microbes are present mainly on mucosal surfaces and are governed mainly by the host, and transient microbes are largely present in the digesta and are influenced mainly by diet (Ringø et al., 2016; Kashinskaya et al., 2018; Legrand et al., 2020). Resident microbes are governed mainly by deterministic processes, and transient microbes are governed mainly by neutral processes (Wilkes Walburn et al., 2019; Heys et al., 2020).

**FIGURE 1:**
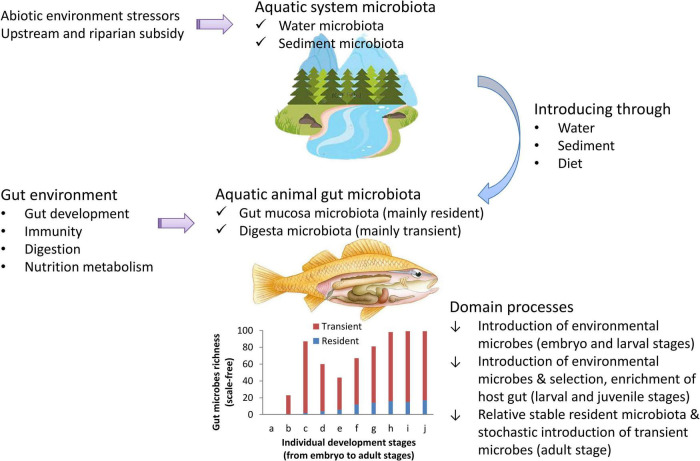
A general framework for understanding aquatic animal gut microbiota dynamics.

Estimating the impacts of the environment on fish gut microbial communities is a key step to better assessing and managing host health and aquatic ecosystem health (Sehnal et al., 2021). Fish gut microbiomes aid in host nutrient absorption, xenobiotic metabolism, energy homeostasis, intestinal development, immune system function, and so on (Butt and Volkoff, 2019; Sehnal et al., 2021). The fish gut microbiota can be significantly affected by various factors, such as host genotype, immunity, pathobiology, diet, ecotype and abiotic environment (Ni et al., 2014; Yan et al., 2016; Vasemägi et al., 2017; Butt and Volkoff, 2019). These effects are partly driven by deterministic selection processes of the host (Yan et al., 2016; Wilkes Walburn et al., 2019; Dvergedal et al., 2020; Xiao et al., 2021), and the other effects are maintained by neutral microbes introducing processes from the environment (Sun et al., 2019; Heys et al., 2020; Le and Wang, 2020). Here, we described the dynamics of the fish gut microbiota in natural waters using a general introducing-then-filtering framework (Figure 1). Environmental factors influence gut microbial communities and then impact the health of hosts and even of ecosystems (Adamovsky et al., 2018; Duperron et al., 2019; Evariste et al., 2019). To assess these impacts, one needs to first assess the effects of environmental factors on gut microbial communities (Adamovsky et al., 2018; Evariste et al., 2019; Duperron et al., 2020). Before assessing the effects of environmental factors on gut microbial communities, one needs to identify the dominant process of how the environment influences gut microbes.

Previous work identified that gut microbial composition was highly influenced by species and diet as well as by the environment (Sullam et al., 2012; Wong and Rawls, 2012; Vasemägi et al., 2017; Adamovsky et al., 2018), and the colonized gut microbiota was governed by both deterministic and neutral processes (Heys et al., 2020). To identify the dominant process by which the environment influences gut microbes, the current study aimed to quantify the colonization of environmental microbes in the fish gut. Following the general introducing-then-filtering framework of gut microbial community dynamics (Figure 1), we hypothesize that, for adult fishes, gut microbial communities are dominated by the introduction of environmental microbes. In other words, in adult fish, gut microbial communities are mainly transient rather than resident. Then, to test our hypothesis, we used a case study to (1) quantify the proportion of gut microbes that shared with environmental microbial community, (2) quantify the proportion of environmental microbes that shared with gut microbial community, (3) quantify the proportion of core (i.e., resident) gut microbes in pan (i.e., whole) gut microbes; and (4) identify the main factors influencing gut microbes by comparing the structure of the water environmental microbial community and the gut microbial community in 10 wild fish populations from the Wuhan section of the Yangtze River.

## Materials and Methods

### Study Area

The Yangtze River is the third longest large river ecosystem in the world, with a length of more than 6300 km and a catchment area of 1.8 × 10^6^ km^2^. From the headwater to the estuary, the river crosses four climatic zones and has an elevation difference of 5400 m. Supported by the diversity of climate, hydrology and habitat, there is rich aquatic biodiversity, with more than 400 fish species and subspecies, of which 45% are endemic (Zhang et al., 2020a). Fishes in the Yangtze River support approximately 60% of inland fish production in China (Zhang et al., 2020b). Driven by various anthropogenic stressors, such as damming, legal overfishing and illegal fishing, water pollution, the reclamation of lakes for farmland, the isolation of lakes from rivers, waterway channel construction and vessel navigation, wild fish stocks have dramatically decreased and fish biodiversity has obviously decreased in past decades (Zhang et al., 2020a,b). In 2019, a 10-year comprehensive fishing ban was implemented in the Yangtze River and its key tributaries (Zhang et al., 2020c). It is estimated that fish stock in the Yangtze River will increase in future annuals (Zhang et al., 2020b). Understanding the mechanism by which environmental stressors affect recovering fish communities via key elements of gut microbes is critical.

### Sampling Procedures

On September 12 to 28 of 2020, we caught the fishes of *Leiocassis crassilabris*, *Leiocassis longirostris*, *Pelteobagrus vachelli*, *Silurus asotus*, *Hypophthalmichthys molitrix*, *Aristichthys nobilis*, *Coreius heterodon*, *Xenocypris argentea*, *Siniperca chuatsi*, and *Coilia brachygnathus* (Table 1) using a floating gill net in a transect of the Yangtze River (N30°34′14′′, E114°38′36′′) located in Shuangliu town, Xinzhou district, Wuhan city, Hubei province, PRC. These fishes were kept in an ice bath and carried to our lab. All individuals were dissected, and then, approximately 0.5 g of the gut (including gut contents) was sampled aseptically. The guts from multiple individuals were pooled into one sample, especially for the small individuals. At least three replicates per species were collected, and the samples were frozen on dry ice and then stored at –80°C until DNA extraction.

**TABLE 1:** The 10 fish species in the current study.

Group label	Species	Order	Family	Habitats	Diets
*L.C.*	*Leiocassis crassilabris*	Siluriformes	Bagridae	Bottom	Oligochaeta, small mollusks, shrimps, little fishes
*L.L.*	*Leiocassis longirostris*	Siluriformes	Bagridae	Bottom	Little fishes, shrimps, aquatic insects
*P.V.*	*Pelteobagrus vachelli*	Siluriformes	Bagridae	Bottom	Aquatic insects, Oligochaeta, shrimps, small mollusks, little fishes
*S.A.*	*Silurus asotus*	Siluriformes	Siluridae	Bottom	Shrimps, little fishes
*H.M.*	*Hypophthalmichthys molitrix*	Cypriniformes	Cyprinidae	Pelagic	Phytoplankton, zooplankton
*A.N.*	*Aristichthys nobilis*	Cypriniformes	Cyprinidae	Pelagic	Zooplankton, phytoplankton
*C.H.*	*Coreius heterodon*	Cypriniformes	Cyprinidae	Bottom	Small mollusks, fish eggs and larvae, phytoclasts
*X.A.*	*Xenocypris argentea*	Cypriniformes	Cyprinidae	Bottom	Bottom attached algae, phytoclasts
*S.C.*	*Siniperca chuatsi*	Perciformes	Serranidae	Pelagic	Fishes, shrimps
*C.B.*	*Coilia brachygnathus*	Clupeiformes	Engraulidae	Pelagic	Little fishes, shrimps

*Data from the China Animal Scientific Database (http://www.zoology.csdb.cn/).*

Along with fish catching, a 1.5-L surface water sample was collected using a 1.5-L sterilized bottle (rinsed three times with sampling water) each day. Because keeping the samples cool can reduce the rate of eDNA decay and is a convenient and efficient method for conserving eDNA samples (Sales et al., 2019), water samples were transported in an ice bath (0°C) to the lab each day. To obtain the eDNA of the microbial communities (Eichmiller et al., 2016; Li et al., 2018), water samples (with purified water used as a negative control) were filtered by using 0.2-μm membrane filters (JinTeng, Tianjin, PRC) to obtain the eDNA sample in the laboratory. Subsequently, the filter membranes of each water sample were placed in a 50-mL sterilized centrifuge tube. The samples were transported at –20°C (in a dry ice bath) and stored at –80°C until DNA extraction.

### DNA Extraction and Sequence Analysis

Here, we analyzed the microbial communities using metabarcoding of 16S rRNA, restricted the amplified fragment length to 300–500 bp and selected the primer 338F/806R (Yang et al., 2020, 2021). Our samples (both gut samples and water eDNA samples) were processed by Shanghai Majorbio Bio-pharm Technology Co., Ltd. (Shanghai, China), and our data were analyzed on the free online Majorbio Cloud Platform^1^. The technical details of DNA extraction, sequencing and data processing were described in our previous work (Yang et al., 2020, 2021). The data that support the findings of this study have been deposited into CNGB Sequence Archive (CNSA) of China National GeneBank DataBase (CNGBdb) with accession number CNP0002410, CNP0002411.

### Statistical Analysis

The raw sequence data of each sample was analyzed on the Majorbio Cloud Platform, and the operational taxonomic unit (OTU), the sequence number of each OTU, and the taxonomic features of each sample were obtained. Subsequently, the sample size, sequence number, OTU number and other sequence characteristics of each sample of ten species group and water environment group were calculated. Additionally, the rarefaction curves of each species group and water environment group at the OTU level were calculated. The OTUs accumulation curves of each species group and water environment, group were calculated. Alpha diversity analysis, including analysis of the Sobs and Chao richness index and Invsimpson and Shannon diversity index at the OTU level, was conducted to reveal the variation in all group samples. NMDS (Non-metric multidimensional scaling) analysis, PLS-DA (Partial Least Squares Discriminant Analysis) and ANOSIM (analysis of similarities) on the samples of all species groups were processed at OTU level to reveal the dissimilarity among the samples of all groups. We defined shared microbial OTUs between fish gut microbes and water environmental microbes as the OTUs appearing in both fish gut microbial community and water environmental microbial community. In each fish gut sample and in each species group, the proportion of gut microbes that shared with water environmental microbial community at the OTU level was calculated to reveal the influence of water environmental microbes on fish gut microbial community. The proportion of water environmental microbes that shared with gut microbial community of each species group at the OTU level and species level was calculated to reveal the introduction capacity of water environmental microbes into the fish gut. The proportion of core gut microbes (indicating resident gut microbes) in the pan gut microbes (indicating whole-gut microbes, including resident and transient gut microbes) at the OTU level and species level was calculated to identify the dominant process in the gut microbial community. The core gut microbes are identified as the OTUs/species that are detected in all gut microbial samples of a fish species group, and were estimated using the core analysis (the curve of shared microbial OTUs/species number vs. sample number) and the power regression equation fitting. The pan gut microbes are identified as the OTUs/species that are detected in any gut microbial sample of a fish species, and were estimated using the pan analysis (the curve of total microbial OTUs/species number vs. sample number) and the power regression equation fitting of new OTUs/species number for a next sample. Community composition analysis and a community heatmap showing the 200 most abundant species in the bacterial communities of each species group and water environment group were conducted to reveal the similarity of groups based on their microbial communities.

## Results

### Samples Characteristics

**FIGURE 2:**
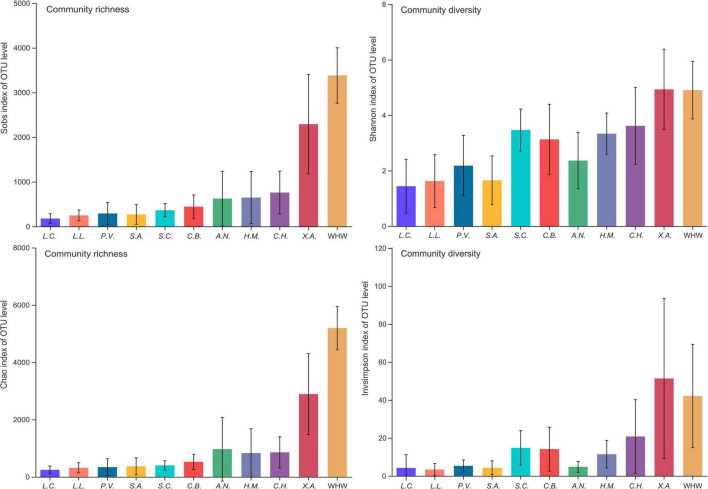
The microbial community richness and diversity of each group. Ten fish species groups of *Leiocassis crassilabris* (*L.C.*), *Leiocassis longirostris* (*L.L.*), *Pelteobagrus vachelli* (*P.V.*), *Silurus asotus* (*S.A.*), *Hypophthalmichthys molitrix* (*H.M.*), *Aristichthys nobilis* (*A.N.*), *Coreius heterodon* (*C.H.*), *Xenocypris argentea* (*X.A.*), *Siniperca chuatsi* (*S.C.*), *Coilia brachygnathus* (*C.B.*) and one water environment sample group at the Wuhan transect of the Yangtze River (WHW).

A total of 10,477,369 clean sequences were obtained from 168 adult fish gut samples of the 10 species groups and 13 samples of the 1 water environment group (more details in Supplementary Table 1). A total of 15,640 OTUs were detected among these sequences, which belonged to 3 kingdoms, 67 phyla, 198 classes, 500 orders, 860 families, 1977 genera and 4393 species (more details in Supporting Information 2_OTU Table). The rarefaction curve of each sample at the OTU level indicated that the sequence depth was almost sufficient (Supplementary Figure 1). The OTUs accumulation curves of each group indicated that OTUs would increase along with the increase of samples (Supplementary Figure 2). The microbial community richness and diversity of each sample were highly variable among species groups and water environment group (Figure 2 and Supplementary Figure 3). These variations were at both the individual sample level and the species group level (Figure 2 and Supplementary Figure 3). The dominant genera of gut microbial community were cetobacterium, *Clostridium*, *Acinetobacter*, *Mycoplasma*, *Ralstonia*, *Plesiomonas*, an unclassified genus in peptostreptococcaceae, an unclassified genus in vibrionaceae, and an unclassified genus in clostridiaceae (more details in Supplementary Figure 4 and Supporting Information 3_Dominant Microbial Genera). There was obvious dissimilarity between fish gut microbial community and water environment microbial community (Supplementary Figures 5–7).

### Influence of Water Environmental Microbes on Fish Gut Microbes

**FIGURE 3:**
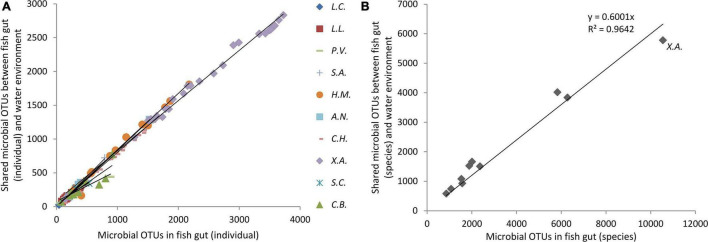
Proportion of gut microbes for each fish individual **(A)** and each fish species **(B)** that shared with water environmental microbial community at the OTU level. Ten fish species groups of *Leiocassis crassilabris* (*L.C.*), *Leiocassis longirostris* (*L.L.*), *Pelteobagrus vachelli* (*P.V.*), *Silurus asotus* (*S.A.*), *Hypophthalmichthys molitrix* (*H.M.*), *Aristichthys nobilis* (*A.N.*), *Coreius heterodon* (*C.H.*), *Xenocypris argentea* (*X.A.*), *Siniperca chuatsi* (*S.C.*), and *Coilia brachygnathus* (*C.B.*).

For each fish individual, nearly 80% (78.8∼81.5%, CI = 0.95) of the fish gut microbes (at the OTU level) were shared with water environmental microbes, although there was individual variation (Figure 3A). The proportions of the gut microbes (at the OTU level) that shared with water environmental microbes in *Siniperca chuatsi* and *Coilia brachygnathus* (both are pelagic piscivorous fish) individuals were lower than those in individuals of other fish species, with a value of only approximately 74%. For fish species, more than 60% of fish gut microbes (at the OTU level) were shared with water environmental microbes, except for *Xenocypris argentea* (only 55%), which is a bottom scraper (Figure 3B and Supplementary Figure 8).

### Introduction of Water Environmental Microbes Into the Fish Gut

**FIGURE 4:**
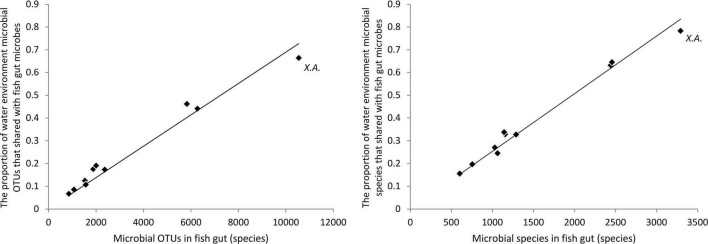
Proportion of water environmental microbes shared with gut microbes. *X.A.* refers to the fish species group of *Xenocypris argentea*.

The proportion of water environmental microbes shared with gut microbes was in direct proportion to fish gut microbes at both the OTU level and the species level (Figure 4). In other words, in a fish with richer fish gut microbes, a higher capacity of water environmental microbes were introduced into the fish gut microbial community. For a species, a higher capacity to take and keep microbes drives more water environmental microbes to be introduced into the gut. For the bottom scraper, *Xenocypris argentea*, compared with the community richness of gut microbiota, there was a relatively low proportion of water environmental microbes introduced into the fish gut (Figure 4). Comparing the microbial OTUs and species between total fish gut microbes and total water environment microbes, nearly 79% of OTUs and 89% of species of water environment microbes were introduced into the fish gut.

### Resident Microbes in Fish Guts

**FIGURE 5:**
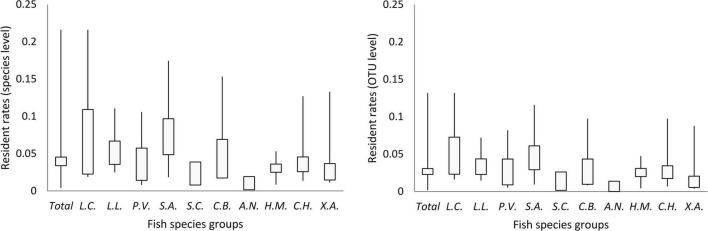
The proportion of core (resident) gut microbes in the gut microbial community of each fish species group (CI = 0.95). Ten fish species groups of *Leiocassis crassilabris* (*L.C.*), *Leiocassis longirostris* (*L.L.*), *Pelteobagrus vachelli* (*P.V.*), *Silurus asotus* (*S.A.*), *Hypophthalmichthys molitrix* (*H.M.*), *Aristichthys nobilis* (*A.N.*), *Coreius heterodon* (*C.H.*), *Xenocypris argentea* (*X.A.*), *Siniperca chuatsi* (*S.C.*), and *Coilia brachygnathus* (*C.B.*).

Less than 5% of OTUs (in 86% of individuals) and species (in 77% of individuals) were the core (resident) microbes of the corresponding species group, although there were individual variations and species differences (Figure 5 and Supplementary Figure 9). Moreover, in the pan (including resident and transient) microbes of each species group, less than 1% of OTUs and species were identified to be core (resident) microbes, although there was a species difference (Table 2). The individuals and species of *Xenocypris argentea* and *Aristichthys nobilis* had the lowest gut microbial residence rates (Figure 5, Supplementary Figure 9, and Table 2). The individuals and species of *Leiocassis crassilabris*, *Leiocassis longirostris*, and *Silurus asotus* had the highest gut microbial residence rates (Figure 5, Supplementary Figure 9, and Table 2).

**TABLE 2:** The proportion of core (resident) gut microbes in the pan (including resident and transient) gut microbes of the corresponding fish species group.

Group label	OTU level %	Species level %
*L.C.*	0.54	0.74
*L.L.*	0.40	0.85
*P.V.*	0.21	0.39
*S.A.*	0.37	0.96
*H.M.*	0.20	0.43
*A.N.*	0.07	0.15
*C.H.*	0.22	0.55
*X.A.*	0.12	0.55
*S.C.*	0.21	0.40
*C.B.*	0.24	0.61

*Ten fish species groups of Leiocassis crassilabris (L.C.), Leiocassis longirostris (L.L.), Pelteobagrus vachelli (P.V.), Silurus asotus (S.A.), Hypophthalmichthys molitrix (H.M.), Aristichthys nobilis (A.N.), Coreius heterodon (C.H.), Xenocypris argentea (X.A.), Siniperca chuatsi (S.C.), and Coilia brachygnathus (C.B.).*

### Gut Bacterial Community Similarity Among Fish Species Groups

**FIGURE 6:**
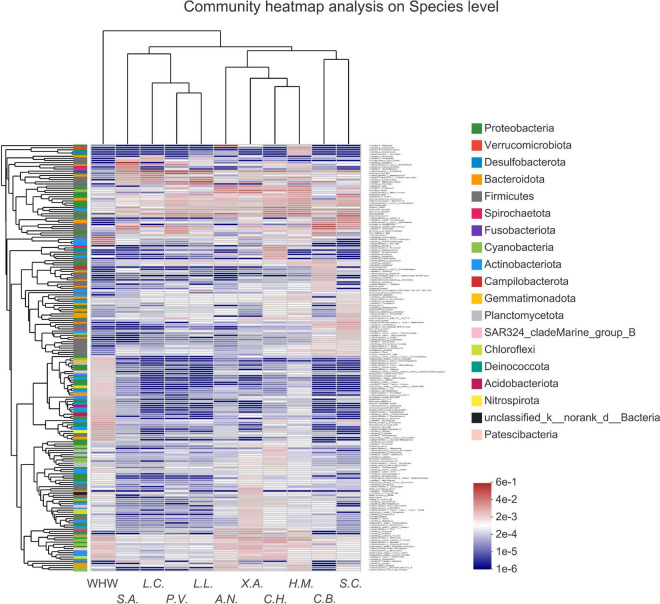
Community heatmap of the 200 most abundant species in the bacterial communities of each fish species group and water environment group. Ten fish species groups of *Leiocassis crassilabris* (*L.C.*), *Leiocassis longirostris* (*L.L.*), *Pelteobagrus vachelli* (*P.V.*), *Silurus asotus* (*S.A.*), *Hypophthalmichthys molitrix* (*H.M.*), *Aristichthys nobilis* (*A.N.*), *Coreius heterodon* (*C.H.*), *Xenocypris argentea* (*X.A.*), *Siniperca chuatsi* (*S.C.*), *Coilia brachygnathus* (*C.B.*) and one water environment sample group at the Wuhan transect of the Yangtze River (WHW).

The result of PLS-DA on the microbial community of all samples showed that the samples of *Leiocassis crassilabris*, *Leiocassis longirostris*, *Pelteobagrus vachelli*, *Silurus asotus* (bottom predators), and *Siniperca chuatsi* (pelagic predator) had the highest similarity, and then *Coilia brachygnathus* (pelagic predator) and *Aristichthys nobilis* (pelagic planktivore) (Supplementary Figure 7). The samples of *Hypophthalmichthys molitrix*, *Coreius heterodon*, and *Xenocypris argentea* (phytophagy-related fishes) were obviously dissimilar with those samples (Supplementary Figure 7). Moreover, the community heatmap analysis showed that the species group of *Siniperca chuatsi* and the species group of *Coilia brachygnathus* (both are pelagic piscivorous fish) had similar gut microbial communities (Figure 6). The species groups *Hypophthalmichthys molitrix*, *Coreius heterodon*, *Xenocypris argentea*, and *Aristichthys nobilis* (all feed on plants more or less) had similar gut microbial communities (Figure 6). The species groups *Leiocassis longirostris*, *Pelteobagrus vachelli*, *Leiocassis crassilabris*, and *Silurus asotus* (all bottom predators) had similar gut microbial communities (Figure 6).

## Discussion

### Most Microbes in the Gut Are Transient

The gut microbes of fish are classified as resident, i.e., those that colonize the host’s epithelial surface or are associated with the microvilli, or as transient, i.e., those that are associated with digesta or are present in the lumen (Ringø et al., 2016; Legrand et al., 2020). A previous work indicated that most gut microbes were transient (Heys et al., 2020). Along with host development, some transient microbes are filtered by the host gut environment (Heys et al., 2020). In the current work, we quantified the proportion of transient gut microbes at both the OTU and the species levels in 10 adult wild fishes according to estimates of the core gut microbes and pan gut microbes. In approximately 80% of individuals, more than 95% (at both the OTU and the species levels) were transient microbes (Figure 5 and Supplementary Figure 9). For each fish species, among the microbes introduced into the gut, more than 99% (at both the OTU and the species levels) were transient microbes (Table 2). Of course, there were weak individual variations and interspecific differences (Figure 5, Supplementary Figure 9, and Table 2). In other words, the gut microbial dynamic of wild adult fish was governed mainly by stochastic processes.

### Transient Microbes Originated From Environmental Microbes

Transient gut microbes are driven by stochastic processes, such as the random introduction of environmental microbes to the gut environment without obvious adaptation (Heys et al., 2020), which causes the environment to shape a similar gut microbial structure (Sun et al., 2020). Water, as a microbe matrix, provides a source for the gut microbiota of host animals (Kuang et al., 2020; Sun et al., 2020; Sehnal et al., 2021). In the current work, water samples had the highest microbial community richness (Figure 2 and Supplementary Figure 3). In most individuals, approximately 80% of fish gut microbes (at the OTU level) were shared with water environmental microbes, except for individuals of *Siniperca chuatsi* and *Coilia brachygnathus* (approximately 74%) (Figure 3A). For fish species groups, more than 60% of fish gut microbes (at the OTU level) were shared with water environmental microbes (Figure 3B). Considering that OTUs would increase along with the increase of samples (Supplementary Figure 2), for some species, the proportion of shared microbes maybe overestimated in our results.

Moreover, sediment is an important microbe matrix (Kuang et al., 2020; Zhou et al., 2021) and is an important source of intestinal bacteria (Sun et al., 2019, 2020). A previous work showed that approximately 72.5 and 48.2% of gut microbial OTUs from 17 cultured species were shared with sediment and water samples, respectively (Sun et al., 2019). In the current work maybe, influenced by the introduction of sediment environmental microbiota, only 55% of the gut microbiota (at OTU level) of the bottom scraper *Xenocypris argentea* was shared with water environmental microbes (Figure 3B and Supplementary Figure 8). Perhaps a main part of the gut microbiota of the bottom scraper was shared with sediment environmental microbes.

### Introduction of Environmental Microbes Relies on Feeding Habits

Environmental microbes are introduced into the host gut, always with the diet (Kuang et al., 2020; Zhang et al., 2021a; Zhou et al., 2021). Therefore, there were significant differences among transient gut microbes of omnivorous, zoobenthivorous, zooplanktivorous, and piscivorous fishes (Kashinskaya et al., 2018). For pelagic organisms, especially pelagic filter-feeding fish, the water microbiota contributes to the formation of the main gut microbiota (Kuang et al., 2020). For benthonic organisms, especially bottom detritus feeders, sediment is the main source of the bacteria contributing to the formation of the gut microbial community (Zhang et al., 2021a; Zhou et al., 2021). In other words, for certain fish, only certain environmental microbes can be introduced into the gut. Consequently, gut (transient) microbiomes always cluster based on diet, regardless of species (Xing et al., 2013; Kashinskaya et al., 2018; Pratte et al., 2018; Riiser et al., 2020). In reef fish, the gut microbiomes of herbivores are similar to those of omnivore microbiomes and then carnivore microbiomes (Pratte et al., 2018). Due to the propensity of omnivorous fish to consume small invertebrates and plants, the gut microbes of omnivorous fish are similar to planktonic and invertebrate microbial communities (Sullam et al., 2012; Sehnal et al., 2021). In the current work, the gut microbes of *Leiocassis crassilabris*, *Leiocassis longirostris*, *Pelteobagrus vachelli* (bagridae, bottom predators) had the same dominant genera (Supplementary Figure 4). Chloroplast was one of the dominant parts in the guts of *Hypophthalmichthys molitrix*, *Aristichthys nobilis*, *Coreius heterodon*, *Xenocypris argentea* (phytophagy-related fishes) (Supplementary Figure 4). PLS-DA result showed that bottom predators had the highest similarity, and then was similar with the pelagic predators, the pelagic planktivore, and the phytophagy-related fishes (Supplementary Figure 7). The community heatmap analysis showed that two pelagic piscivorous fishes were clustered as a group, four more-or-less-plants-feeding fishes were clustered as a group, and four bottom predators were clustered as a group (Figure 6). These results verified that the gut microbes of these wild fishes relied mainly on feeding habits.

Driven by the introduction of environmental microbes that rely on feeding habits, omnivores and filter feeders always have high gut microbial community richness and diversity. Considering that sediment is an important microbe matrix (Kuang et al., 2020; Zhou et al., 2021), bottom omnivores, scrapers and detritus feeders have a high gut microbial community richness and diversity (Sun and Xu, 2021). In the current work, the gut microbiota of *Xenocypris argentea* (a bottom scraper) had the highest community richness, followed by the gut microbiota of *Coreius heterodon*, *Hypophthalmichthys molitrix*, and *Aristichthys nobilis* (Figure 2 and Supplementary Figure 2). The gut microbiota of *Leiocassis crassilabris*, *Leiocassis longirostris*, *Pelteobagrus vachelli*, *Silurus asotus*, *Siniperca chuatsi*, and *Coilia brachygnathus* had similar and the lowest community richness (Figure 2 and Supplementary Figure 2). Because that *Siniperca chuatsi* and *Coilia brachygnathus* were pelagic predators, and that *Leiocassis crassilabris*, *Leiocassis longirostris*, *Pelteobagrus vachelli*, and *Silurus asotus* were bottom predators, they clustered as two groups (Figure 6).

### Monitoring and Managing Environmental Microbes to Conserve Aquatic Animals

The gut microbiota is tightly linked to host health and profoundly influenced by the environmental microbiota (Butt and Volkoff, 2019; Sehnal et al., 2021). The current work estimated that in approximately 80% of individuals, more than 95% of microbes were transient (Figure 5 and Supplementary Figure 9), and approximately 80% of the microbial composition was shared with the environmental microbial community (Figure 3A). Moreover, fish gut microbes can be dispersed to the surrounding water (Burns et al., 2017; Troussellier et al., 2017). Monitoring and managing the environmental microbial community is a form of indirectly monitoring and managing aquatic animal gut microbial communities and indirectly monitoring and managing aquatic animal health. As environmental microbial communities respond rapidly (even in hours or days) to environmental disturbances (Païssé et al., 2010; Landesman and Dighton, 2011; Sehnal et al., 2021), monitoring and managing environmental microbial communities could be an active process to conserve aquatic animals.

Considering that the introduction of environmental microbes relies on feeding habits and that certain environmental microbes are introduced into certain fish gut, environmental microbial community monitoring should include different habits, such as surface water, middle-layer water, bottom water, sediment, plants and detritus. Because of the gut microbial functional profiles shaped by the intestinal environment (Sun and Xu, 2021), microbial structure would vary with hosts and environments. Would this impact the effectiveness of monitoring and managing the environmental microbial community to conserve aquatic animal health? We need more research. Of course, environmental microbial community monitoring and management support not only aquatic animal conservation but also ecosystem health conservation (Sehnal et al., 2021). Because of technical progress in high-throughput sequencing and microbiota data analysis, environmental microbiota would be useful and general indicators of host and ecosystem health in the future (Sehnal et al., 2021).

### Quantification Variation of Gut Microbial Community Characteristics

The quantification of gut microbial community characteristics maybe vary with samples, seasons, environments and hosts. In adult fish, resident microbes are present mainly on mucosal surfaces and are governed mainly by the host, and transient microbes are largely present in the digesta and are influenced mainly by diet (Ringø et al., 2016; Kashinskaya et al., 2018; Legrand et al., 2020). Therefore, the gut samples with contents would detect more transient microbes than the gut samples without contents do. Along with the seasonal variation of diet supply and environmental microbial community in aquatic ecosystem (Element et al., 2020; Yang et al., 2021), the microbial quantity and assemblage that was introduced into gut would vary with seasons. Because that environmental pollutants affect gut microbiota composition (Evariste et al., 2019; Hua et al., 2021; Zhang et al., 2021b,c), the richness, diversity and structure of gut microbiota would vary with different water environmental conditions. Shaped by host gut environment (Sun and Xu, 2021), the proportion of resident/transient microbes would vary with host species.

In the current work, we take the gut samples (including gut contents) in 10 fish populations from the Wuhan section of the Yangtze River with a good water environmental condition on September. The results showed that in most individuals, approximately 80% of gut microbial OTUs were shared with the water environmental microbial community, and more than 95% of microbe species (OTUs) in the gut were transient. For fish species, more than 99% of microbe species (OTUs) that were introduced into the gut were transient. Nearly 79% of OTUs and 89% of species of water environmental microbes could be introduced into the fish gut. There were interspecific differences and individual variations. Therefore, all these quantitative descriptions of gut microbes were approximate results at the definite research design condition rather than accurate quantities for general conditions. We hoped that our results could provide a reference for future studies.

The proportions of fish gut microbes that shared with water environment microbial community in previous studies range from 4.6 to 48.2% (Borsodi et al., 2017; Sun et al., 2019; Kuang et al., 2020; Meng et al., 2021). In the current work, the proportion is approximately 80% for each wild adult fish individual, more than 60% for each species group (except for *Xenocypris argentea*, only 55%). It seems that these variations are enormous, but understandable. In the case that derived the proportion of 4.6% from one water sample and one hindgut content sample of *Aristichthys nobilis*, the microbial OTUs in water and in hindgut contents respectively were 254 and 65 (Borsodi et al., 2017). In the case with 6 gut parallel samples and 9 water samples that was conducted in an unfed aquaculture reservoir, nearly 13% of gut microbes of *Hypophthalmichthys molitrix* and *Aristichthys nobilis* were shared with the water (Kuang et al., 2020). In the case with 3 gut parallel samples and 3 water samples that was conducted in aquaculture pond systems, approximately 36% of gut microbes of *Hypophthalmichthys molitrix* and *Aristichthys nobilis* were shared with the water (Meng et al., 2021). In the case with 17 gut samples, 12 water samples and 13 sediment samples that was conducted in seawater aquaculture pond systems, approximately 48% of the gut microbes were shared with the water and nearly 73% of the gut microbes were shared with the sediment (Sun et al., 2019). Comparing with the current work, there were relative lower water microbial richness and gut microbial richness in these previous studies (Borsodi et al., 2017; Sun et al., 2019; Kuang et al., 2020; Meng et al., 2021), which was probably driven by insufficient parallel samples or definite aquaculture environment and then resulted in the underestimated or low proportion of gut microbes that shared with water environment microbial community. It should be noted that the shared gut microbes proportion for each species group (Supplementary Figure 8) maybe overestimated in our results, because that the gut microbes richness of part of fish species groups was seriously underestimated (Supplementary Figure 2).

## Conclusion

In adult fish, for most individuals, more than 95% microbes (at both the OTU and the species levels) were transient, and the gut microbiota dynamic was governed mainly by stochastic introduction processes. Following the continuous random microbes introduced from the environment into the gut, in each individual, approximately 80% of fish gut microbes (at the OTU level) were shared with water environmental microbes, except for individuals of *Siniperca chuatsi* and *Coilia brachygnathus* (approximately 74%). As the microbe introduction process was tightly linked to fish feeding habits, only a certain portion of environmental microbes could be introduced into the fish gut, and fishes with similar feeding habits had a similar gut microbial structure. To sensitively monitor and actively manage aquatic animal health under anthropogenic and natural disturbance, we could use the water and sediment environmental microbial community as an early warning index.

## Data Availability Statement

The datasets that were generated for this study can be found in the China National GeneBank Sequence Archive (CNSA, https://db.cngb.org/cnsa/) of the China National GeneBank database (CNGBdb) under accession numbers CNP0002410 and CNP0002411.

## Author Contributions

HY: conceptualization, methodology, investigation, data curation, formal analysis, visualization, writing—original draft preparation, writing—reviewing and editing. JW: investigation, data curation, formal analysis, writing—reviewing and editing. HD: conceptualization, funding acquisition, project administration, supervision, validation, writing—reviewing and editing. HZ and JL: investigation. QW: funding acquisition, project administration, resources, supervision, validation, writing—reviewing and editing. All authors contributed to the article and approved the submitted version.

## Conflict of Interest

The authors declare that the research was conducted in the absence of any commercial or financial relationships that could be construed as a potential conflict of interest.

## Publisher’s Note

All claims expressed in this article are solely those of the authors and do not necessarily represent those of their affiliated organizations, or those of the publisher, the editors and the reviewers. Any product that may be evaluated in this article, or claim that may be made by its manufacturer, is not guaranteed or endorsed by the publisher.

## Footnotes

1
www.majorbio.com
