# Supplementary material for: Quantifying the Colonization of Environmental Microbes in the Fish Gut: A Case Study of Wild Fish Populations in the Yangtze River
Source: Front Microbiol. 2022 Feb 17;12:828409. doi: 10.3389/fmicb.2021.828409 (PMC8891936; doi:10.3389/fmicb.2021.828409)
Supplement: Supplementary file 1 [file Data_Sheet_1.docx]

## Supporting Information 1

### Tables (with captions)

Table S1 Sample size, sequence characters and OTU number of each species group and water environment group

| Species/water environment group | Group babel | Sample size | Seq_num | Base_num | Mean_length | Min_length | Max_length | OTU |
| --- | --- | --- | --- | --- | --- | --- | --- | --- |
| *Leiocassis crassilabris* | *L.C.* | 12 | 655916 | 268952003 | 410.0403 | 262 | 510 | 1071 |
| *Leiocassis longirostris* | *L.L.* | 13 | 681569 | 280674835 | 411.8069 | 262 | 521 | 1532 |
| *Pelteobagrus vachelli* | *P.V.* | 9 | 541788 | 226238205 | 417.577 | 218 | 485 | 1569 |
| *Silurus asotus* | *S.A.* | 15 | 857423 | 361047559 | 421.0845 | 201 | 477 | 1884 |
| *Hypophthalmichthys molitrix* | *H.M.* | 28 | 1442964 | 603625683 | 418.3235 | 212 | 521 | 5829 |
| *Aristichthys nobilis* | *A.N.* | 4 | 209582 | 88692804 | 423.189 | 247 | 498 | 2007 |
| *Coreius heterodon* | *C.H.* | 29 | 1648551 | 688330074 | 417.5364 | 239 | 540 | 6274 |
| *Xenocypris argentea* | *X.A.* | 31 | 1958992 | 813850256 | 415.4434 | 207 | 532 | 10545 |
| *Siniperca chuatsi* | *S.C.* | 3 | 150190 | 63046843 | 419.7806 | 239 | 431 | 855 |
| *Coilia brachygnathus* | *C.B.* | 11 | 632153 | 260275845 | 411.7292 | 238 | 517 | 2366 |
| Wuhan water environment | WHW | 13 | 1698241 | 709705588 | 417.9063 | 201 | 534 | 8707 |

### Figures (with captions)


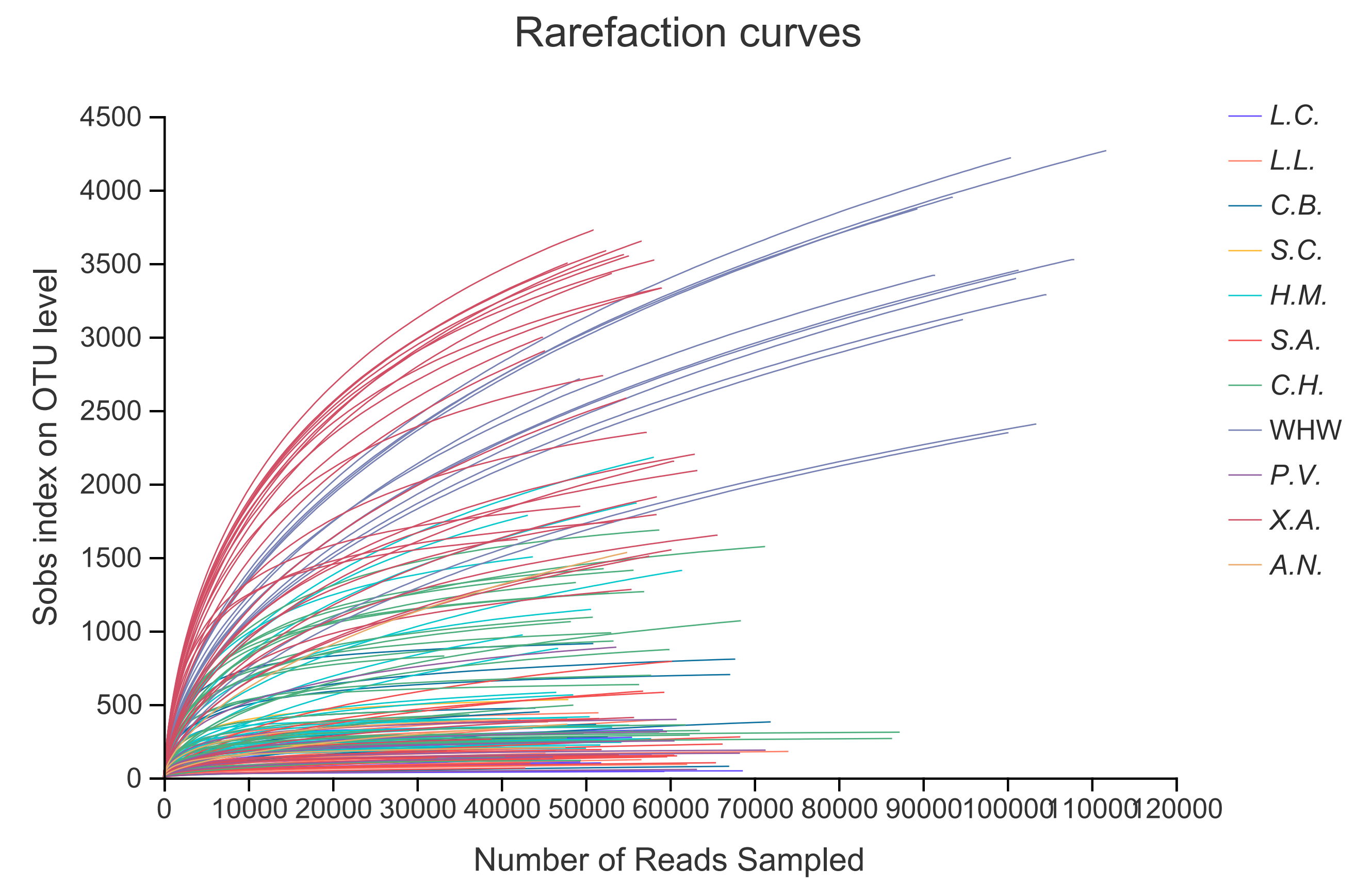


Figure S1 Rarefaction curves of each sample of ten species groups and water environment group at the OTU level

Ten species groups of *Leiocassis crassilabris* (*L.C.*), *Leiocassis longirostris* (*L.L.*), *Pelteobagrus vachelli* (*P.V.*), *Silurus asotus* (*S.A.*), *Hypophthalmichthys molitrix* (*H.M.*), *Aristichthys nobilis* (*A.N.*), *Coreius heterodon* (*C.H.*), *Xenocypris argentea* (*X.A.*), *Siniperca chuatsi* (*S.C.*), *Coilia brachygnathus* (*C.B.*) and one water environment sample group at the Wuhan transect of the Yangtze River (WHW).


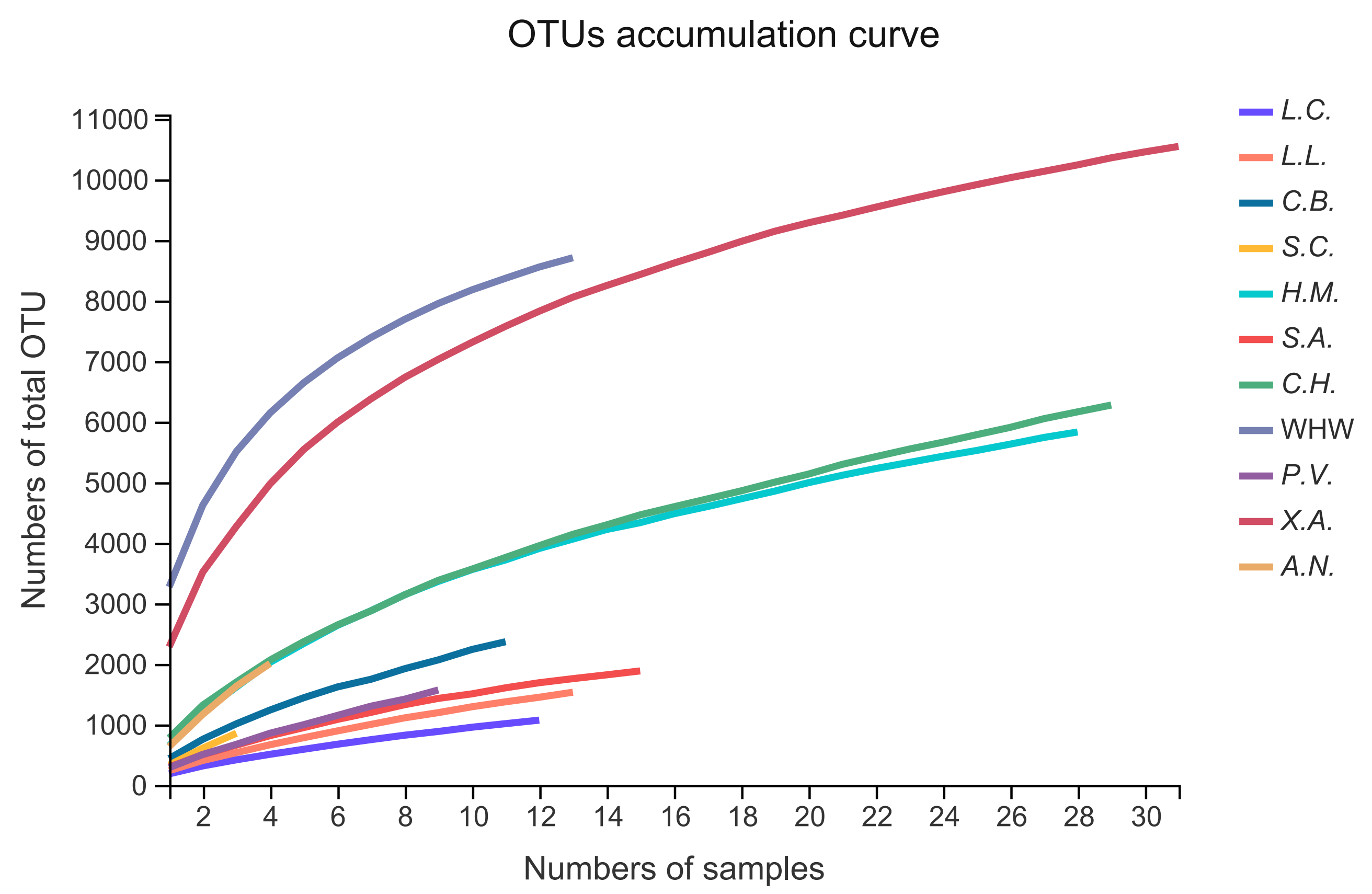


Figure S2 OTUs accumulation curves of each species group and water environment group

Ten species groups of *Leiocassis crassilabris* (*L.C.*), *Leiocassis longirostris* (*L.L.*), *Pelteobagrus vachelli* (*P.V.*), *Silurus asotus* (*S.A.*), *Hypophthalmichthys molitrix* (*H.M.*), *Aristichthys nobilis* (*A.N.*), *Coreius heterodon* (*C.H.*), *Xenocypris argentea* (*X.A.*), *Siniperca chuatsi* (*S.C.*), *Coilia brachygnathus* (*C.B.*) and one water environment sample group at the Wuhan transect of the Yangtze River (WHW).


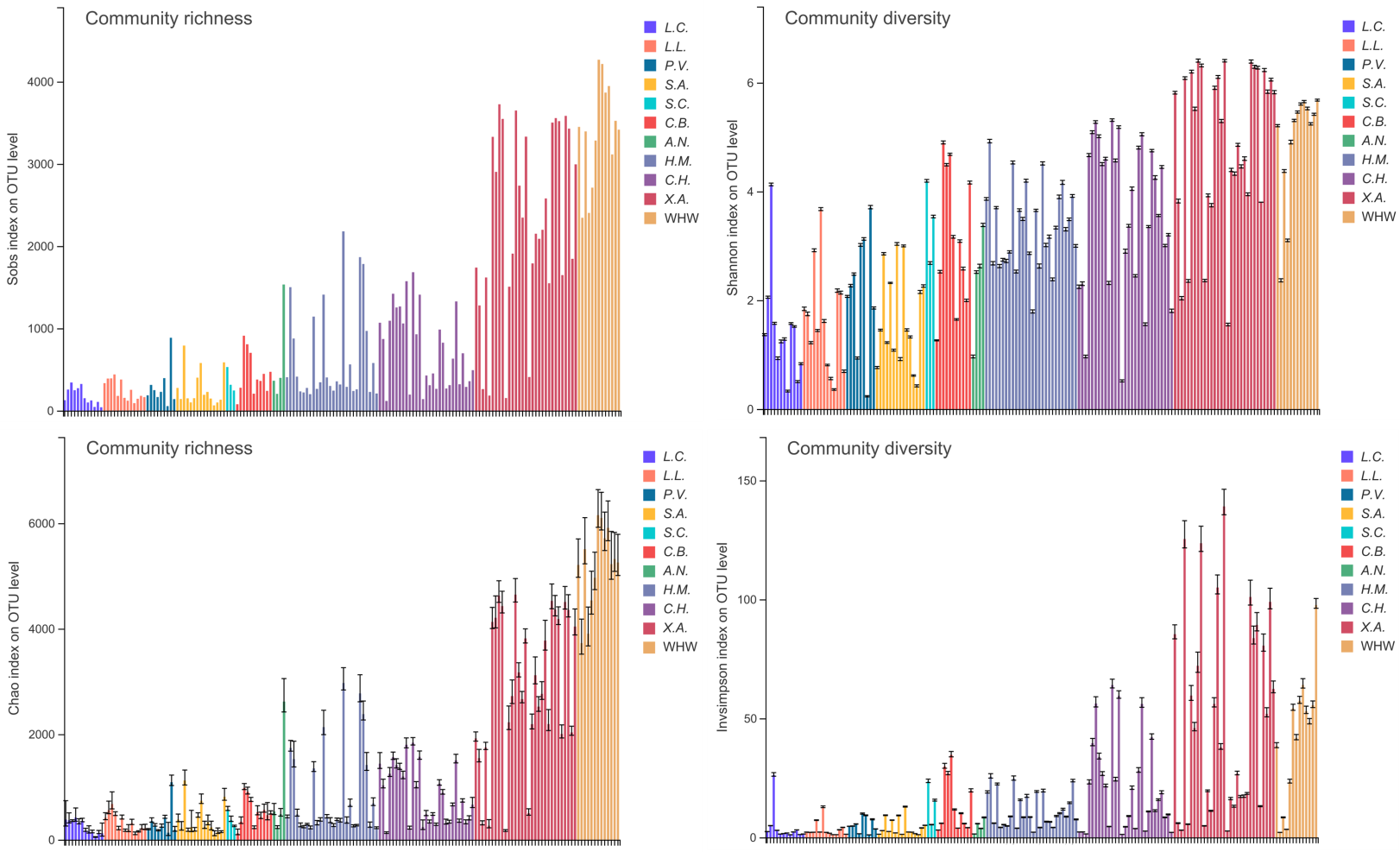


Figure S3 The microbial community richness and diversity of each sample

Ten species groups of *Leiocassis crassilabris* (*L.C.*), *Leiocassis longirostris* (*L.L.*), *Pelteobagrus vachelli* (*P.V.*), *Silurus asotus* (*S.A.*), *Hypophthalmichthys molitrix* (*H.M.*), *Aristichthys nobilis* (*A.N.*), *Coreius heterodon* (*C.H.*), *Xenocypris argentea* (*X.A.*), *Siniperca chuatsi* (*S.C.*), *Coilia brachygnathus* (*C.B.*) and one water environment sample group at the Wuhan transect of the Yangtze River (WHW).


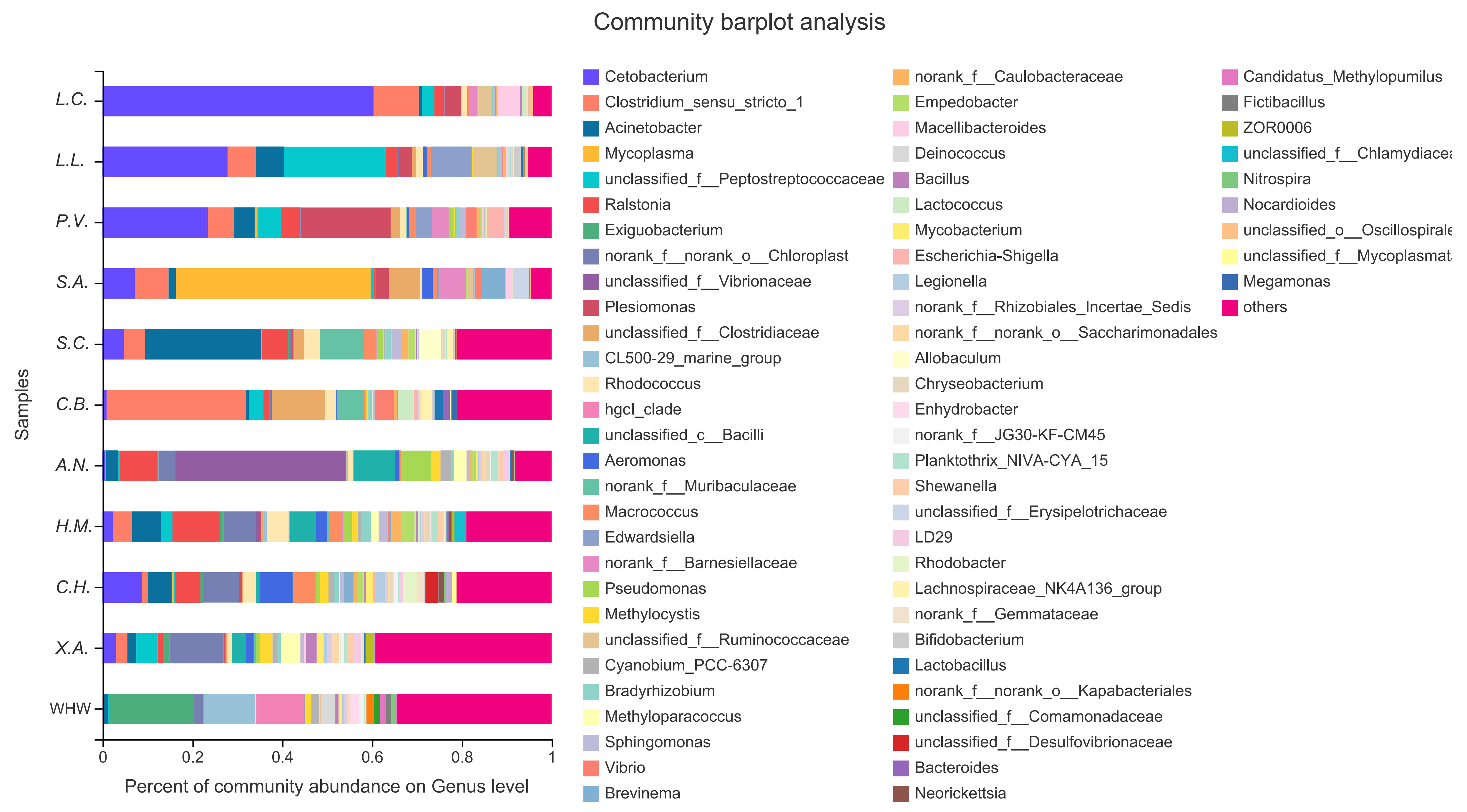


Figure S4 The gut microbial community structure of each species group and water environment group

Ten species groups of *Leiocassis crassilabris* (*L.C.*), *Leiocassis longirostris* (*L.L.*), *Pelteobagrus vachelli* (*P.V.*), *Silurus asotus* (*S.A.*), *Hypophthalmichthys molitrix* (*H.M.*), *Aristichthys nobilis* (*A.N.*), *Coreius heterodon* (*C.H.*), *Xenocypris argentea* (*X.A.*), *Siniperca chuatsi* (*S.C.*), *Coilia brachygnathus* (*C.B.*) and one water environment sample group at the Wuhan transect of the Yangtze River (WHW).


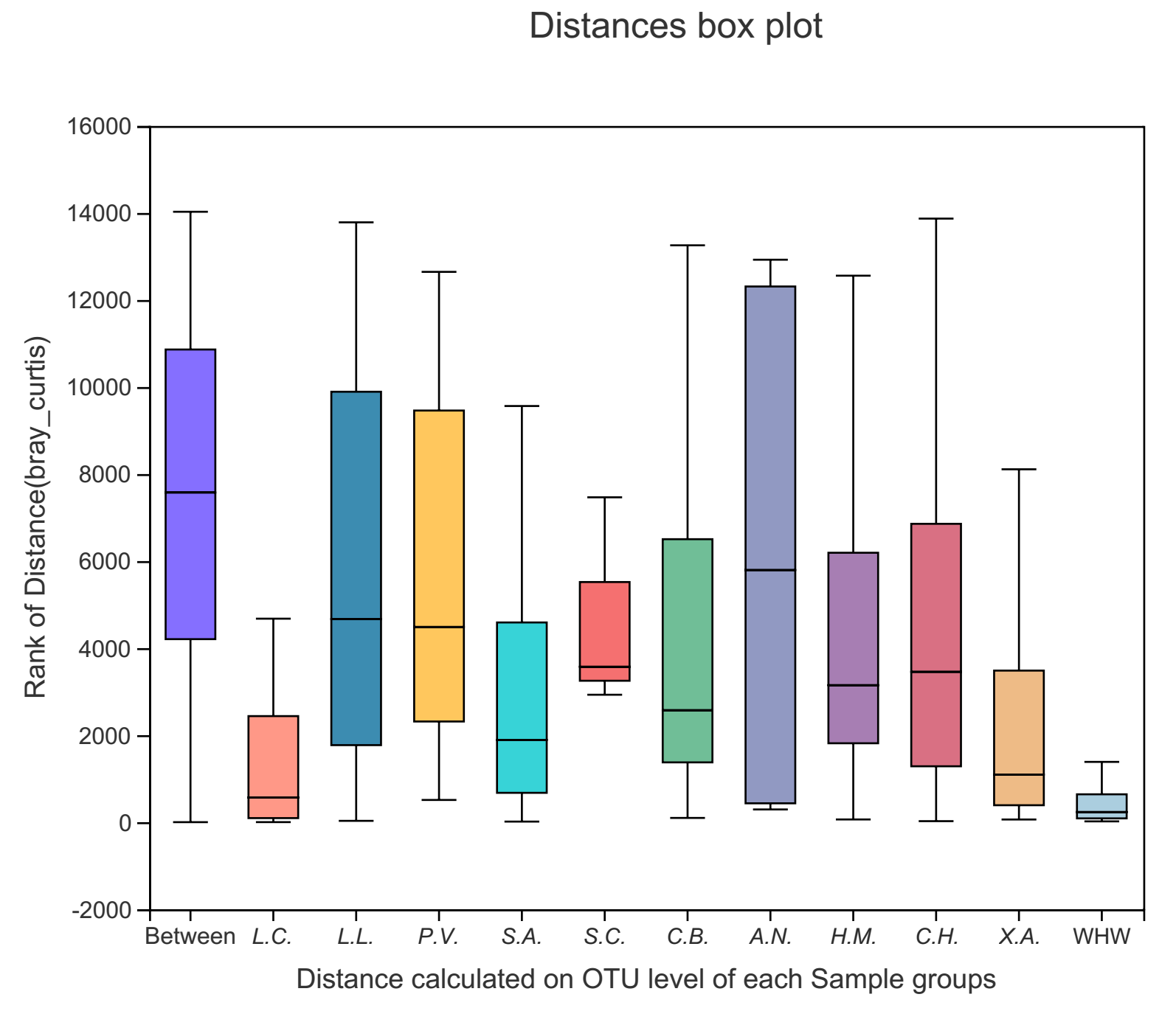


Figure S5 ANOSIM (analysis of similarities) on the groups

Ten species groups of *Leiocassis crassilabris* (*L.C.*), *Leiocassis longirostris* (*L.L.*), *Pelteobagrus vachelli* (*P.V.*), *Silurus asotus* (*S.A.*), *Hypophthalmichthys molitrix* (*H.M.*), *Aristichthys nobilis* (*A.N.*), *Coreius heterodon* (*C.H.*), *Xenocypris argentea* (*X.A.*), *Siniperca chuatsi* (*S.C.*), *Coilia brachygnathus* (*C.B.*) and one water environment sample group at the Wuhan transect of the Yangtze River (WHW).


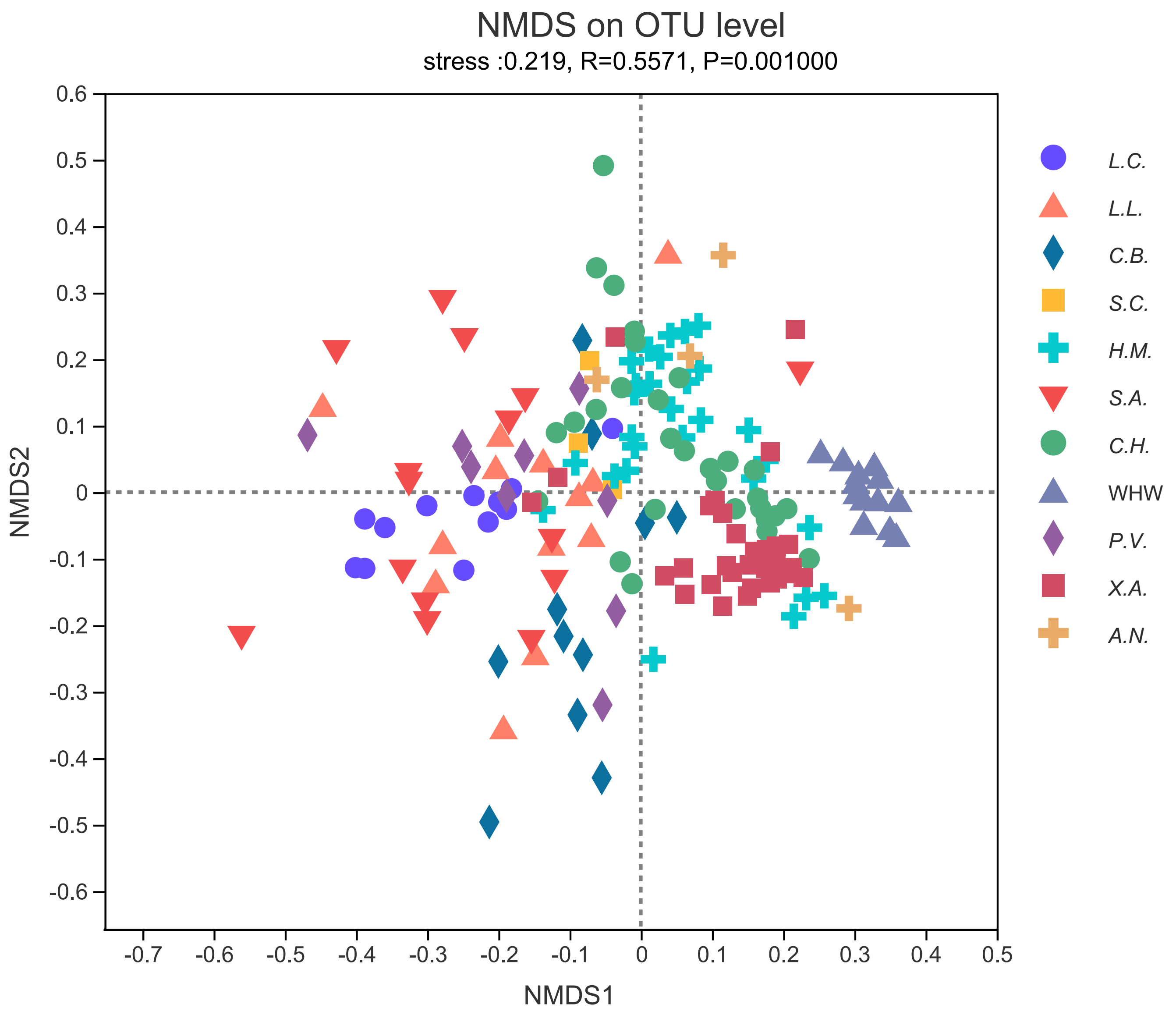


Figure S6 NMDS (Non-metric multidimensional scaling) analysis on the microbial communities of all samples

Ten species groups of *Leiocassis crassilabris* (*L.C.*), *Leiocassis longirostris* (*L.L.*), *Pelteobagrus vachelli* (*P.V.*), *Silurus asotus* (*S.A.*), *Hypophthalmichthys molitrix* (*H.M.*), *Aristichthys nobilis* (*A.N.*), *Coreius heterodon* (*C.H.*), *Xenocypris argentea* (*X.A.*), *Siniperca chuatsi* (*S.C.*), *Coilia brachygnathus* (*C.B.*) and one water environment sample group at the Wuhan transect of the Yangtze River (WHW).


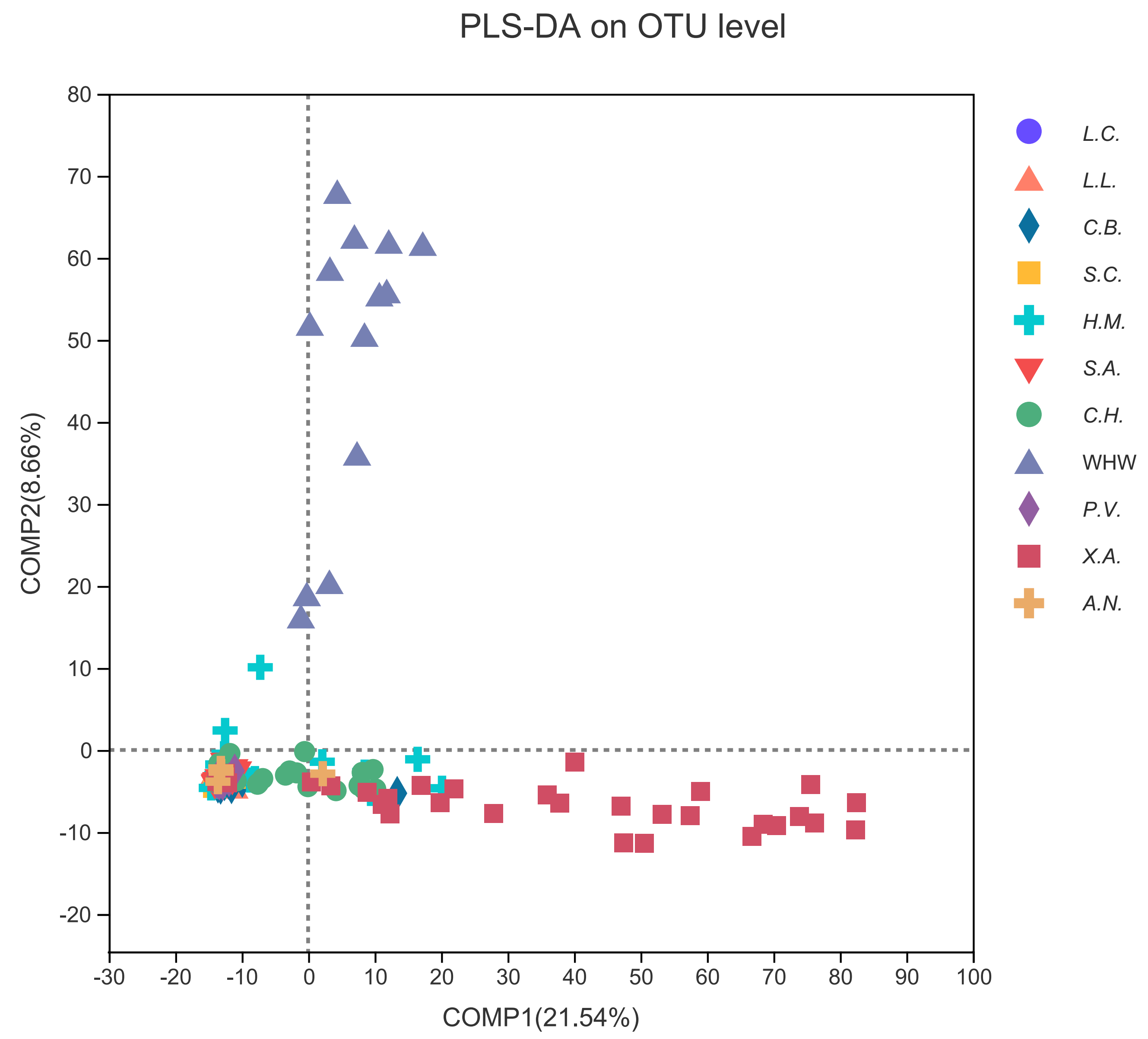


Figure S7 PLS-DA (Partial Least Squares Discriminant Analysis) on the microbial communities of all samples

Ten species groups of *Leiocassis crassilabris* (*L.C.*), *Leiocassis longirostris* (*L.L.*), *Pelteobagrus vachelli* (*P.V.*), *Silurus asotus* (*S.A.*), *Hypophthalmichthys molitrix* (*H.M.*), *Aristichthys nobilis* (*A.N.*), *Coreius heterodon* (*C.H.*), *Xenocypris argentea* (*X.A.*), *Siniperca chuatsi* (*S.C.*), *Coilia brachygnathus* (*C.B.*) and one water environment sample group at the Wuhan transect of the Yangtze River (WHW).


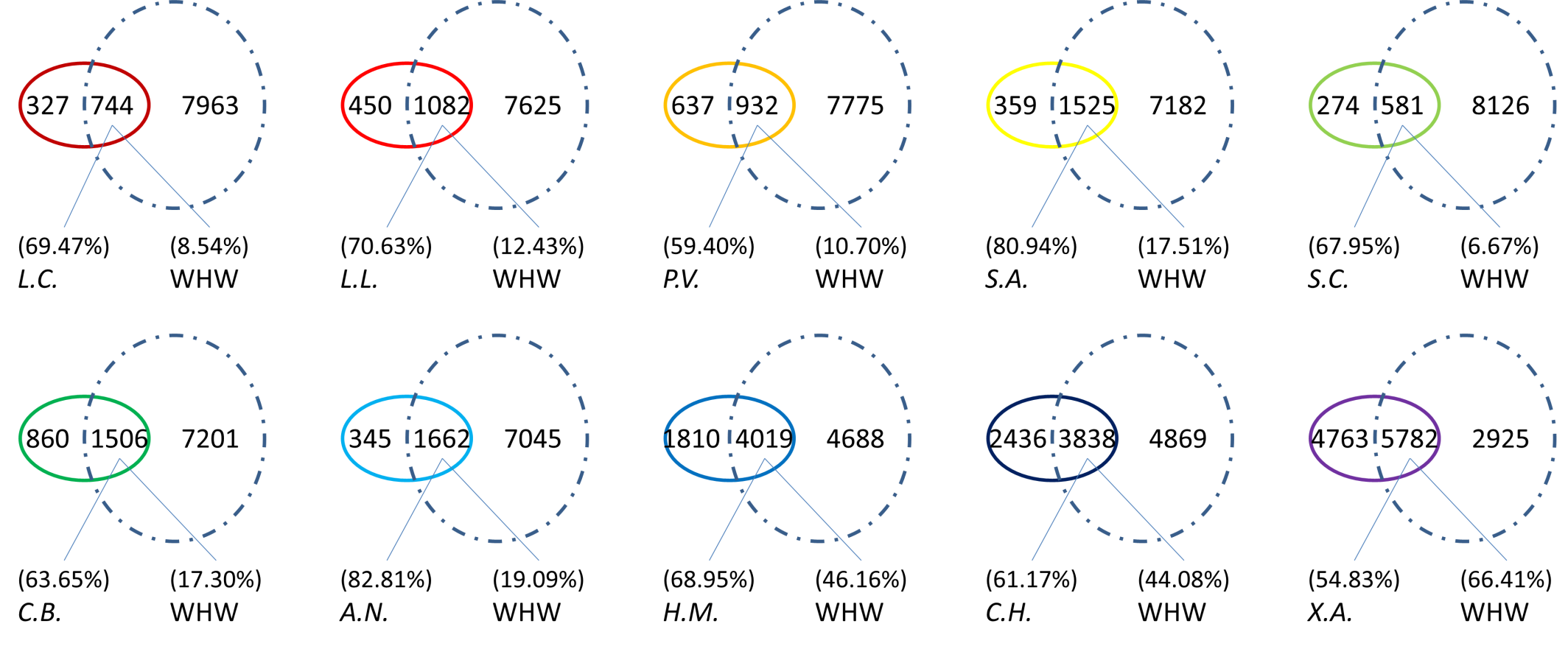


Figure S8 Venn diagrams for shared microbial OTUs between fish gut and water environment

Ten species groups of *Leiocassis crassilabris* (*L.C.*), *Leiocassis longirostris* (*L.L.*), *Pelteobagrus vachelli* (*P.V.*), *Silurus asotus* (*S.A.*), *Hypophthalmichthys molitrix* (*H.M.*), *Aristichthys nobilis* (*A.N.*), *Coreius heterodon* (*C.H.*), *Xenocypris argentea* (*X.A.*), *Siniperca chuatsi* (*S.C.*), *Coilia brachygnathus* (*C.B.*) and one water environment sample group at the Wuhan transect of the Yangtze River (WHW).


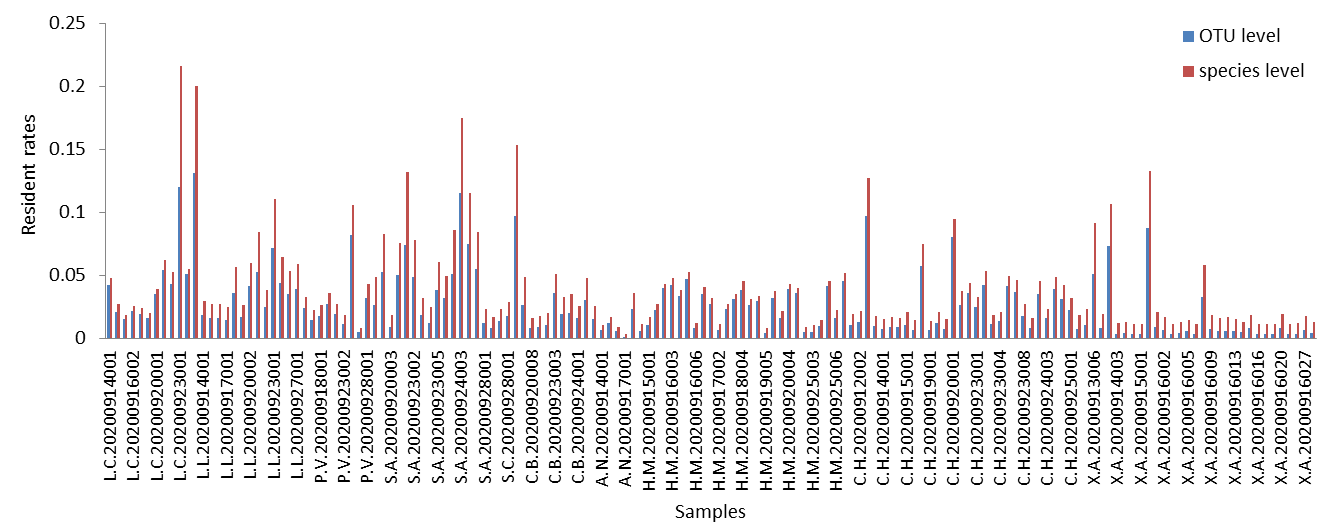


Figure S9 The proportion of core (resident) gut microbes in each individual gut microbial community

Ten species groups of *Leiocassis crassilabris* (*L.C.*), *Leiocassis longirostris* (*L.L.*), *Pelteobagrus vachelli* (*P.V.*), *Silurus asotus* (*S.A.*), *Hypophthalmichthys molitrix* (*H.M.*), *Aristichthys nobilis* (*A.N.*), *Coreius heterodon* (*C.H.*), *Xenocypris argentea* (*X.A.*), *Siniperca chuatsi* (*S.C.*), and *Coilia brachygnathus* (*C.B.*).
